# Severe case of COVID −19 pneumonia complicated by SIADH

**DOI:** 10.1016/j.amsu.2021.103153

**Published:** 2021-12-04

**Authors:** Mohamed Fajri, Meryem Essafti, Rachid Aloua, Youssef Mouaffak

**Affiliations:** a Faculty of Medicine and Pharmacy of Marrakesh, Morocco; b Anesthesia and Critical Care Department, CHU Mohamed VI, Marrakesh, Morocco; c Faculty of Medicine and Pharmacy, Hassan II University of Casablanca, B.P 5696, Casablanca, Morocco

**Keywords:** Hyponatremia, Antidiuretic hormone, The COVID-19 infection, Critical care

## Abstract

**Introduction:**

Hyponatremia is one of the most common water–electrolyte imbalances in the human organism. This disorder is usually secondary to various diseases, including infections. The syndrome of inappropriate secretion of antidiuretic hormone (SIADH) is among causes of hyponatremia in critical care. It can be induced by a variety of conditions, including atypical lung disease.

**Discussion:**

The COVID-19 infection is associated with multiples metabolic disorders. However, hyponatremia associated with SIADH and Coronavirus disease 2019 (COVID-19) is not a usual clinical situation, but it was recently mentioned in a few case reports.

**Conclusion:**

We discuss a unique case of an acute symptomatic hyponatremia and SIADH, attributed to COVID-19.

## Highlights

• The COVID-19 infection is associated with multiples metabolic disorders.
• Hyponatremia is one of the most common water–electrolyte imbalances in the human organism.
• It is usually secondary to various diseases.
• The syndrome of inappropriate secretion of antidiuretic hormone is among causes of hyponatremia in critical care.

## 1 Introduction

At the end of 2019, a cohort of patients with pneumonia of unknown etiology declared in Wuhan, China, announces the start of the COVID-19 pandemic. On June 03, 2020, a total of 6,530,472 confirmed cases (including 385,347 deaths) have been listed by the World Health Organization [1].

The COVID-19 infection is due to a severe acute respiratory syndrome coronavirus-2 (SARS-CoV-2). Although COVID-19 appears to have greater contagiousness, its mortality is lower than SARS and MERS-COV. Its clinical manifestations are dominated by respiratory symptoms which can range from a simple cough or sore throat to a severe ARDS (acute respiratory distress syndrome), in addition to systemic and metabolic complications following a cytokinetic storm. Among observed metabolic disorders, inappropriate secretion of antidiuretic hormone (SIADH) is a syndrome characterized by the prolonged release of antidiuretic hormone (ADH) in the absence of osmotic or non-osmotic stimuli or by enhanced renal action of ADH. It accounts for approximately 50% of all diagnosed cases of hyponatremia [2].

We report a case of hyponatremia due to SIADH in a patient hospitalized in intensive care for COVID-19 infection.

## 2 Case report

**Fig. 1:**
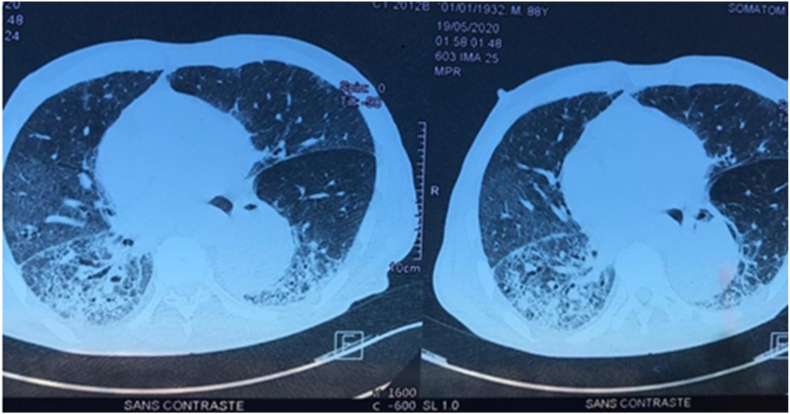
Chest-CT results showing bilateral ground-glass opacities extending over 50%.

A 88 years-old male patient with no particular past medical history, has been admitted to the intensive care for the management of acute respiratory distress following COVID-19 infection. On admission, the patient was conscious with a Glasgow score of 15, elevated heart rate at 110 beats/minute, normal blood pressure and polypneic at 30 cycles/minute with signs of respiratory struggle (subcostal and intercostal retractions), Pulsed oxygen saturation increased from 80% to 90% on 6 L/min oxygen mask. Diagnosis was confirmed by a positive RT-PCR nasopharyngeal swab and suggestive chest-CT results showing bilateral ground-glass opacities extending over 50% (Fig. 1).

Initial biological severity assessment showed: lymphopenia at 500/mm³, elevated levels of serum ferritin (1170ng/ml), CRP (172mg/l), LDH (600UI/l), cardiac troponin (30ng/l), D-dimers(3000 μg/l) and fibrinogen (5.3g/l). Whereas hepatic and renal functions were normal along with serum electrolytes (natremia at 135 mmol/l, serum potassium at 3.9 mmol/l and calcemia at 90 mmol/l). Cardiac assessment was unremarkable with normal electrocardiogram and QT interval. Management consisted in applying non-invasive ventilation with positive expiratory pressure. A medical treatment based on chloroquine and azithromycin, corticosteroids (methylprednisolone 120 mg twice a day on the first day and 60 mg twice a day from the 2nd to the 5th day), prophylactic high dose of anticoagulant therapy(tinzaparin 9000ui/day), large spectrum antibiotics (ceftriaxon 2g/day), vitamin therapy based on vitamin C 2g/day and Zinc supplementation 220mg/d. The patient's development was marked 5 days after hospitalization by confusion and temporospatial disorientation with headaches and generalized hypotonia without convulsions. A biological assessment revealed a severe hyponatremia at 123 mmol/liter with a normal hematocrit at 45% and normal glucose levels. Renal and hepatic functions remained normal (serum urea at 0.31g/l and creatine at 5.6mg/l, ALAT at 39 UI/l ASAT at 38 UI/l, alkaline phosphatases: 50UI/l, Gamma-Glutamyl transferase: 40 UI/l, albumin level: 40 g/liter).

Two check-ups two days apart showed the persistence of hyponatremia 123 → 126 → 124 with normal renal and hepatic check-ups. Given the persistence of hyponatremia in our patient, inappropriate secretion of DHA was suspected. To clarify this diagnosis, our patient was in euvolemia without signs of dehydration, vomiting or diarrhea, no diuretic intake, the diuresis was kept at 0.6ml/kg/hour. No signs of heart failure: no edema, no rales at auscultation. Echocardiography was performed showing no cardiomegaly, normal filling pressures and left ventricular ejection fraction. No signs of hepatic cytolysis or cholestasis. A urinary ionogram and thyroid screening were completed showing elevated natriuresis up to 135mmol/l,a low plasma osmolality at 250 mosm/kg,a high urine osmolality at 300mosm/kg with normal hepatic, renal and thyroid results. The treatment was based on fluid restriction with good clinical and biological progress: a gradual increase in serum sodium concentration from 122 to 135 over 5 days. The patient remained in intensive care for 15 days and was later transferred to the COVID-19 ward.

## 3 Discussion

The SARS-Cov2 infection that appeared in China in December 2019 (COVID-19) constitutes a major challenge for intensive care units due to a larger number of admissions. Almost 5% of infected patients require invasive ventilation and a significant proportion of these patients remain in intensive care for a long period and, require a more specific approach to avoid and treating complications linked to this disease such as thromboembolic events, renal failure, secondary bacterial infection, and cardiovascular complications [3]. The severity of COVID-19 disease mainly affects elderly patients with co-morbidities such as high blood pressure, diabetes. obesity and chronic renal failure [4]. Pulmonary involvement in the form of pneumonia is widespread in people infected with COVID-19, as shown in our case and the literature [5]. SIADH is characterized by dilution hyponatremia (<135mmol/l), increased urinary sodium level (>20mmol/l), high urine osmolality (>100 mOsm/kg) compared to plasma osmolality (<280 mOsm/kg), in euvolemic patients taking no diuretics, with normal cardiac, hepatic, renal, adrenal and thyroid functions [6,7]. SIADH can be induced by a variety of conditions, including atypical lung disease. Syndrome of inappropriate secretion of the antidiuretic hormone (SIADH) accounts for approximately 50% of all diagnosed cases of hyponatremia. The mechanism of SIADH in pneumonia is not well established; however, animal models have determined the role of low intravascular fluid volume and low osmolality of extracellular fluid [8,9]. In the context of intravascular volume depletion, baroreceptors in the carotid sinus and aorta activate the renin-angiotensin system. This, in turn, triggers the secretion of non-osmotic DHA mediated by baroreceptors [10,11]. In the event of a decrease in intravascular osmolality, the osmoreceptors activate and increase the secretion of ADH [12]. Emotional, physical, or psychological stress and pain associated with infections (such as COVID-19) stimulate the hypothalamohypophyseal axis, leading to the release of ADH. Alternatively, stress activates cortical neurons, which stimulate the hypothalamus to secrete ADH [13]. In addition, a lung injury induced by pneumonia can lead to inadequate ventilation-perfusion. This mismatch leads to compensatory hypoxic pulmonary vasoconstriction leading to inadequate filling of the left atrium. As a result, a decrease in the stretching of the left atrium and an increase in the secretion of DHA occur [13]. The marked rise in inflammatory cytokines is described in COVID-19; leading in some cases to a cytokine storm [[14], [15], [16]]. This increase in cytokines can cause SIADH via two mechanisms. First, inflammatory cytokines, such as IL-6, can directly stimulate the non-osmotic release of DHA [17]. Second, these cytokines can damage lung tissue and alveolar cells that can induce SIADH via the hypoxic pulmonary vasoconstriction pathway, as previously described [18]. The hyponatremia of euvolemic dilution must be necessarily differentiated from the hyponatremia due to the salt loss syndrome (Table 1).

**Table 1:** Comparison between SIADH and salt loss syndrome.

Parameters	SIADH	Salt loss syndrome
Extracellular volume	Increased	Decreased
Diuresis	Increased	Decreased
Hematocrit	Normal	Increased
Albuminemia	Normal	Increased
Uricemia	Normal	Decreased
Serum Potassium	Normal	Increased

During the salt loss syndrome; there is a hypovolemia induced hypersecretion of the natriuretic factor responsible for the negative sodium balance, hemoconcentration, and marked hypovolemia. Crystalloid intake is the basic treatment for this syndrome.

## 4 Conclusion

This discussion invites a study to explore the association between secretion of DHA and the levels of inflammatory cytokines, measured in patients COVID-19 infection (with and without pulmonary involvement). This will advance our knowledge of the SIADH mechanisms with further clinical implications. At the time of the pandemic, when medical resources are limited, any additional clues leading to the diagnosis of Covid-19 can be invaluable. Based on this limited evidence, we suggest sorting patients with hyponatremia during this pandemic as a high probability of Covid-19; these patients should be prioritized for testing and isolation whenever possible.

## Ethical approval

Written informed consent was obtained from the patient for publication of this case report and accompanying images. A copy of the written consent is available for review by the Editor-in-Chief of this journal on request.

## Sources of funding

The authors declared that this study has received no financial support.

## Author contribution

Fajri Mohamed: Corresponding author writing the paper.

Youssef mouaffak: Correction of the paper.

## Consent

Written informed consent was obtained from the patient for publication of this case report and accompanying images. A copy of the written consent is available for review by the Editor-in-Chief of this journal on request.

## Registration of Research Studies

1. Name of the registry:
2. Unique Identifying number or registration ID:
3. Hyperlink to your specific registration (must be publicly accessible and will be checked):

## Guarantor

Fajri mohamed.

## Provenance and peer review

Not commissioned, externally peer reviewed.

## Declaration of competing interest

Authors of this article have no conflict or competing interests. All of the authors approved the final version of the manuscript.
